# Evaluation of a Program to Screen Patients in Community Pharmacies for Opioid Misuse and Accidental Overdose

**DOI:** 10.5888/pcd19.220028

**Published:** 2022-07-14

**Authors:** Elizabeth Skoy, Oliver Frenzel, Heidi Eukel, Emily Lothspeich, Jayme Steig, Mark Strand, Amy Werremeyer

**Affiliations:** 1 North Dakota State University School of Pharmacy, Fargo, North Dakota; 2 North Dakota State University School of Public Health, Fargo, North Dakota

## Abstract

**Introduction:**

Community pharmacies nationwide have adopted new strategies to combat the opioid epidemic. One strategy to prevent opioid misuse and accidental overdose is patient screening to identify those at risk. The purpose of our study was to determine whether such screening in community pharmacies led pharmacy personnel to intervene with patients at risk and to describe the proportion of patients they identified as at risk.

**Methods:**

We implemented the Opioid and Naloxone Education (ONE) program in North Dakota to give community pharmacies and pharmacists training and tools to provide preventive screening for opioid misuse and accidental overdose before dispensing a prescribed opioid. Data were collected and analyzed from September 15, 2018, through May 15, 2021, to evaluate overall patient risk characteristics for opioid misuse and accidental overdose.

**Results:**

Of 8,217 patients screened, 3.9% were identified as at high risk for opioid misuse, and 18.3% at risk for accidental overdose. Nearly 1 of 3 screenings (31.7%) indicated opioid medication use in the past 60 days. Pharmacists delivered 1 or more risk-factor–dependent interventions to 41.1% of patients in the study. Following screening, naloxone dispensing in pharmacies increased to 6 times the national average.

**Conclusion:**

Pharmacy-based patient screening for risk of opioid misuse and accidental overdose led to risk-dependent interventions targeted to individual patients. The tools and risk-dependent interventions applied in the ONE program increased patient awareness of opioid risks and ways to reduce risk. Future studies should examine long-term outcomes, including reduction in overdose, treatment of opioid use disorder, and reduced opioid-related acute care.

**Summary**

**What is already known on this topic?**

Pharmacists have a crucial role in the prevention of opioid misuse and accidental overdose. As initiatives to reduce opioid risks are undertaken, patient receipt of opioid interventions at pharmacies is necessary.

**What is added by this report?**

A preventive approach to dispensing opioids in community pharmacies can be targeted to the individual patient’s risk.

**What are the implications for public health practice?**

The pharmacist has an important role in upstream prevention of opioid misuse and overdose. Our data highlight the effectiveness of linking screening with targeted, risk-dependent interventions to identify and intervene with patients at risk.

## Introduction

In October 2017, the Acting Secretary of Health and Human Services declared the opioid epidemic a public health emergency (1). Since then, national pharmacy organizations have banded together to empower pharmacists to take an active and preventive role in addressing the opioid crisis (2,3). In 2018, North Dakota reported that more than half of all drug overdose deaths were attributable to opioids (4). Opioid prescribing rates have consistently declined in the state; however, deaths from overdose of prescribed opioids have continued to rise, with an increase of 8.8% from 2018 through 2019 (5).

Pharmacists in various practice settings have taken steps to prevent opioid misuse and accidental overdose (3,6,7). These steps include evaluating program information on prescription drug monitoring, dispensing naloxone, referring patients for substance use disorder treatment, and enhancing patient and pharmacist education (8–11). Policy changes, such as granting pharmacists the ability to prescribe naloxone and to provide medication treatment of opioid use disorder have assisted in these efforts (12,13). These policy changes have led to improved patient outcomes. For example, administering naloxone to someone experiencing an overdose can reverse the effect of the opioid (14–16). Providing patient education about side effects of opioid medication, including habit formation, is another role pharmacists can play to help prevent opioid-related harm (17,18).

New programs and initiatives implemented in health care settings, including community pharmacies, need to be assessed to understand their impact. The nation’s limited health care resources need to be directed to programs that effectively deliver care and interventions to those who need them most, programs that incorporate primary prevention strategies (19,20).

One approach to preventive health care is patient screening. Screening can facilitate early detection, improve quality of life, and prevent future disease complications (21). Preventive screening by pharmacists could ensure that patients prescribed opioids get the guidance they need to use the medication correctly and safely (20,22,23). Screening for opioid misuse in the community pharmacy is feasible, but information is lacking on the screening interventions themselves and their results (24). Such information is needed to determine whether opioid screening programs are effective and whether patients are receiving interventions targeted to their individual needs. The objectives of our study were to determine the proportion of pharmacy patients based on universal patient screening identified as at risk of opioid misuse and accidental overdose and whether those identified as at risk received targeted interventions. 

## Methods

The Opioid and Naloxone Education Program (ONE; formerly known as ONE Rx) is a statewide program in North Dakota that provides community pharmacies and pharmacists with training and tools to provide preventive universal screening for opioid misuse and accidental opioid overdose for every patient prescribed an opioid prior to dispensing. The program’s design, education for participating pharmacists and technicians, and initial evaluation have been described previously (25–27). The preventive screening is accomplished by using a patient intake form that incorporates the Opioid Risk Tool (ORT) to identify and stratify risk for opioid misuse. ORT collects information on age, sex, family history, and previous substance use (28) and is scored on a scale of 0 to 26 with 0 the lowest risk and 26 the highest risk. To provide the most comprehensive approach to opioid harm reduction, the patient intake form also screens for risk of accidental opioid overdose by collecting information on age, concomitant medications, and comorbidities (26). People with an ORT score of 8 or higher are classified as at high risk for opioid misuse. Risk for accidental overdose is based on the pharmacists’ subjective judgement and evaluation of available patient information (25).

At the time the ONE Program was created, we reviewed multiple opioid risk assessments and decided to incorporate ORT into the patient intake form. We theorized that pharmacists would be able to incorporate ORT into the regular community pharmacy workflow. ORT evaluates preexisting conditions of opioid users distinct from prior pain management strategies. The validation studies for ORT were originally done in a pain management setting, and aberrant behaviors over the 12 months following initiation of opioid use served as the criteria for validating opioid misuse (28). That study demonstrated that 94.4% of patients with an ORT score indicating low risk had no aberrant drug-related behaviors (specificity) whereas 90.9% of those with an ORT score indicating high risk exhibited aberrant behaviors (sensitivity) (28). These validity measures were nearly duplicated in a later validation of a modified version of ORT (29).

All patients prescribed an opioid medication were asked to complete the patient intake form. Most patients completed the form on paper; however, a few pharmacies had patients complete the form on electronic tablets. After the patient completed the patient intake form, the pharmacist evaluated the information provided and then used the program’s triage tool to determine the individual patient intervention needed. Based on patient risk, these interventions may include discussing the patient’s risk of opioid misuse, discussing the availability and benefits of naloxone, dispensing naloxone, contacting the prescriber with concerns, discussing available community support services, and discussing the signs and risk of opioid overdose. In addition to risk-dependent interventions, the pharmacist explains proper disposal of the opioid medication and the option of partially filling the opioid prescription (risk-independent interventions). Each pharmacist-led discussion is tailored to the information the patient provides and that patient’s individual risk. The ONE Program training also offered suggested communication strategies when addressing individual risk. The triage tool and interventions were developed by the ONE Program expert team, which consisted of specialists in psychiatric pharmacy, community pharmacy, public health, and quality improvement and by consulting the health literature.

After the risk-screening process and pharmacy-delivered interventions, the pharmacy staff recorded all patient interventions provided, along with information collected from the patient intake form, in a secure, web-based application called REDCap (www.redcapcloud.com/redcap-cloud-myhealth-ecoa-2?gclid=EAIaIQobChMIhY-xjcql9wIVKvHjBx20rgLMEAAYAiAAEgLGEvD_BwE#eCOA/ePRO), which is designed to support data capture for research studies (30). To reimburse the pharmacy for time spent performing the screening, intervention, and data reporting (which averaged 5 minutes per patient), the pharmacy was paid $20 for each screening (stipends were funded from the grant funding the ONE Program).

Data were collected from September 15, 2018, through May 15, 2021 (32 months) and analyzed by using Microsoft Excel 2019 (Microsoft Corp) to provide descriptive statistics and χ^2^ results to evaluate overall patient risk characteristics for opioid misuse and accidental overdose. We also assessed which risk-dependent interventions were provided by the pharmacist. A χ^2^ test for independence was conducted to examine the relation between patients screening as high-risk versus not at high risk for opioid misuse and the interventions delivered to each group. The North Dakota State University institutional review board approved the study tools and methods.

## Results

During the study period, 8,217 screenings were conducted and documented by participating community pharmacies. The average age of patients undergoing the opioid risk-screening process was 48.8 years. Most were female (54.3%) (Table 1). Patients aged 45 to 64 years were the highest proportion of people screened for opioid risk (36.5%); patients aged 25 to 44 years were the second highest proportion (28.7%). Of all screenings recorded during the reporting period, 3.9% were patients at high risk for opioid misuse (ORT score ≥8), and 18.3% were identified as at risk for accidental opioid overdose. Nearly 1 of 3 screenings indicated opioid medication use in the past 60 days (31.7%). Pharmacists delivered 1 or more risk-dependent interventions to nearly half of all patients screened (41.1%). Pharmacists instructed 85.0% of all patients screened on proper opioid medication disposal, and 4.0% of patients chose to partially fill their opioid prescriptions.

**Table 1:** Patient (N = 8,217) Characteristics and Interventions Delivered, the ONE Program, North Dakota, September 15, 2018–May 15, 2021

Characteristic	n (%)
**Sex**	
Female	4,462 (54.3)
Male	3,755 (45.7)
**Age, y**	
0–17	362 (4.4)
18–24	657 (8.0)
25–44	2,360 (28.7)
45–64	2,997 (36.5)
≥65	1,841 (22.4)
**Patient assessment^a^**	
High risk for opioid misuse (ORT score ≥8^b^)	321 (3.9)
At risk for accidental opioid overdose	1,508 (18.3)
Used an opioid in past 60 days	2,607 (31.7)
Received one or more risk-dependent interventions^c^	3,381 (41.1)
**Prescriber interventions**	
Educated on proper medication disposal	6,945 (84.5)
Partially filled the opioid prescription	329 (4.0)
Patient encounters during which the pharmacy contacted the prescriber with concerns	62 (0.8)
Patient encounters where the pharmacist discussed the benefits and availability of naloxone	2,477 (30.1)
Pharmacist prescribed naloxone	469 (5.7)
Patients dispensed naloxone	174 (2.1)

Abbreviation: ORT, opioid risk tool.

a Excludes 400 patients determined not to be at risk for opioid misuse.

b ORT scale scores range from 0 to 26; 0 to 3 is classified as low risk, 4 to 7 as moderate risk, and 8 or higher as high risk.

c Risk-dependent interventions: discussing opioid use disorder, discussing availability and benefits of naloxone, dispensing naloxone, contacting the prescriber with concerns, discussing community support services, discussing the signs and risk of opioid overdose.

More than 70% of patients at risk for opioid misuse (ORT score ≥8) received 1 or more risk-dependent interventions (Table 2); 43.9% received targeted education about their risk, and 14.6% received information on community support resources to assist in behavioral health treatment. Data analysis indicated a significant (*P* < .001) difference in these 2 categories. Patients who screened as high risk for opioid misuse were more likely to receive an intervention from the pharmacist than those not screening as high risk (Table 2).

**Table 2:** Pharmacist-Delivered Interventions, Patients (n = 321) Identified As At Risk for Opioid Misuse, the ONE Program, North Dakota, September 15, 2018–May 15, 2021^a^

Screening and intervention measure	At high risk, received intervention (n = 321)^b^	Not at high risk, received intervention (n = 7,896)^c^
Provided education and counseling on opioid use disorder	141 (43.9)	1,638 (20.7)
Provided information on community support services	47 (14.6)	306 (3.9)
Provided information on risk of opioid misuse	39 (12.1)	194 (2.5)
Provided information on the availability and benefits of naloxone	164 (51.1)	2,313 (29.3)
Dispensed naloxone	29 (9.0)	145 (1.8)
Contacted patient’s prescriber because of concerns	14 (4.4)	48 (0.6)
Delivered one or more risk-dependent interventions^d^	225 (70.1)	3,156 (40.0)

Abbreviation: ORT, opioid risk tool.

a Values are n (%) unless otherwise indicated. The difference between at risk and not at risk was significant for all screening and intervention measures at *P* < .001, determined by χ^2 ^test.

b ORT score ≥8. ORT scale scores range from 0 to 26; 0 to 3 is classified as low risk, 4 to 7 as moderate risk, and 8 or higher as high risk.

c ORT score <8. ORT scale scores range from 0 to 26; 0 to 3 is classified as low risk, 4 to 7 as moderate risk, and 8 or higher as high risk.

d Risk-dependent interventions: discussing opioid use disorder, discussing availability and benefits of naloxone, dispensing naloxone, contacting the prescriber with concerns, discussing community support services, discussing the signs and risk of opioid overdose.

Of the 8,217 patients screened, 1,508 (18.3%) were identified by the pharmacist as at risk for accidental opioid overdose (Table 3). Nearly half (43.3%) received pharmacist counseling discussing signs and symptoms of opioid overdose, and 81.2% received 1 or more of the risk-dependent interventions. In addition, 67.2% received information on the availability and benefits of naloxone, and 1 of 4 at risk for accidental opioid overdose was prescribed naloxone (26%). More than 10% of patients at risk for overdose were dispensed naloxone with the opioid prescription at the pharmacy. Of patients screening as at risk for opioid overdose, those indicating opioid use in the past 60 days were more than 3 times more likely to receive consultation about the benefits of naloxone (OR, 3.4) and 5 times more likely to have naloxone dispensed (OR, 5.01) compared with patients screened as at risk for opioid overdose who had not used an opioid in the past 60 days. χ^2^ analysis indicated a significant difference between interventions delivered to patients who screened as at risk for accidental opioid overdose versus those screening as not at risk (*P* < .001). Patients screening as at risk for opioid overdose were significantly more likely to receive a harm-reduction intervention from the pharmacist than those who screened not at risk (Table 3).

**Table 3:** Pharmacist-Delivered Interventions for Patients Identified as At Risk for Accidental Opioid Overdose, the ONE Program, North Dakota, September 15, 2018–May 15, 2021^a^

Screening and intervention measure	At risk, received intervention (n = 1,508)	Not at risk, received intervention (n = 6,709)
Counseled about risk of overdose	654 (43.4)	253 (3.8)
Provided information on the availability and benefits of naloxone	1,014 (67.2)	1,454 (21.7)
Dispensed naloxone	153 (10.1)	21 (0.3)
Contacted patient’s prescriber because of concerns	46 (3.1)	16 (0.2)
Provided information on community support services	188 (12.5)	165 (2.5)
Provided information on risk of opioid misuse	185 (12.3)	47 (0.7)
Provided information on one or more risk-dependent interventions^b^	1,225 (81.2)	2,142 (31.9)

a Values are n (%) unless otherwise indicated. The difference between at risk and not at risk was significant for all screening and intervention measures at *P* < .001, determined by χ^2 ^test.

b Risk-dependent interventions: discussing opioid use disorder, discussing availability and benefits of naloxone, dispensing naloxone, contacting the prescriber with concerns, discussing community support services, discussing the signs and risk of opioid overdose.

## Discussion

Screening the right people in a setting that captures the highest possible number of people at risk for a disease or condition who would benefit from screening is essential when implementing risk-screening interventions (22). Identifying people at risk at a time when they are most likely to receive recommendations based on their own personalized risk profile is also important (22). Furthermore, screening everyone with an opioid prescription and making intervention decisions based on objective measures may help overcome the stigma that might otherwise prevent pharmacists or patients from discussing opioid-related risks.

The ONE Program has capitalized on the integration of ORT and an objective measure for overdose risk assessment to screen patients receiving an opioid prescription. The ONE Program was voluntary for pharmacies willing to implement the screening program and for patients completing the intake form; however, an evaluation was conducted to establish the perception of both patients and pharmacists who used the program; 94% of pharmacists reported that patients never or less than 25% of the time felt the screening was offensive or an invasion of privacy (31). In addition, most pharmacists reported that ONE Program screening improved communication, opportunities for interventions, and patient safety and well-being (31). The interventions are indicated based on risk stratification so that only the intended audience receives appropriate interventions. Implementing risk-stratification screening along with risk-dependent interventions allows the health care system to align need with outcomes.

Notably, the national naloxone dispensing rate in 2018 at community pharmacies for patients at risk for overdose was 1.5% (32). In the ONE Program, of patients at risk for accidental opioid overdose (n = 1,508), 10.1% were dispensed naloxone, which is more than 6 times the national average. This number suggests that pharmacists were able to use objective screening information and program-designed training and interventions to further justify a need for naloxone for each opioid patient. Other methods to increase naloxone availability include co-prescribing mandates and opt-out naloxone dispensing. The effectiveness and implementation strategies of these methods vary by pharmacy workflow, state laws, and cost of naloxone (33,34). Research has shown that increasing pharmacy access to naloxone increases the probability of its use, and providing screening and intervention is one approach to increasing naloxone acceptance rates (35,36).

The summarized screening results collected through the ONE Program show a need for risk assessment. With over 8,000 screenings collected, evidence is now available to further support program implementation in a wider population and other health care settings. Almost 4% of patients in our study (n = 321) were at high risk for opioid misuse with an ORT score greater than 8. This statistic would have gone unknown, with over 300 patients potentially receiving an opioid prescription and no risk-dependent intervention to address their risk. With more than 30% of patients having taken an opioid in the past 60 days, this shows a need for screening independent of duration of use of the opioid prescription.

As a prediction tool to determine who will advance to aberrant opioid use behaviors, assessments of ORT have mixed findings (37,38). However, the close association of the variables included in ORT with high-risk use of opioids has been confirmed (39). The standard against which ORT was validated was aberrant opioid misuse behaviors in the 12 months after obtaining the prescribed medication, so it continues to be appropriate to use as a proxy in the ONE Program for risk for opiate use disorder (40). However, the authors continue to assess its utility.

Although community pharmacy integration of opioid risk screening in daily operations is encouraged, associated interventions linked to risk assessment are lacking (24). It is important to determine whether risk assessment leads to positive health outcomes and less health care spending. The ONE Program has stratified risk categories for more than 8,000 patients and determined pharmacist-delivered interventions for various groups of patients. Pharmacist-delivered education and objective risk stratification and triage contributed to targeted risk-level interventions.

Our study had some limitations. The ability of patient screening to predict which patients will go on to experience opioid misuse or overdose cannot be evaluated on the basis of available data. A challenge for pharmacy-led interventions is that community pharmacy software is often not connected to a central electronic patient health record. This produces a few challenges. For one, it creates a barrier for exchange of risk and screening information between clinicians and pharmacists. However, the lack of health records is also a reason why pharmacies should perform their own risk assessment. In addition, without pharmacies having access to health records, it is not possible to determine whether the pharmacist-delivered interventions resulted in improved health outcomes or a reduction in health care expenditures. Future research considerations should include a comparison of the ONE Program process in opioid risk screening with a control group receiving standardized care when receiving a prescribed opioid medication in the pharmacy. Having access to additional health information could also allow the pharmacist to look at other risk-contributing factors such as social determinants of health.

ONE Program screening numbers decreased at the peak of COVID-19-related shutdowns and throughout the COVID-19 pandemic. Future research could include how COVID-19 affected the ONE Program and changes in risk assessment. In addition, although pharmacy technicians were provided education and training for the ONE Program, their impact on the program has not yet been evaluated. Finally, our results cannot be generalized beyond pharmacies in North Dakota, where our study took place.

ONE Program’s community pharmacy–based patient screening for risk of opioid misuse and accidental opioid overdose led to targeted risk-dependent interventions for those screened. The tools and risk-dependent interventions applied in the program led to increases in patient education on opioid risks and ways to reduce risk. Patients screened for risk of opioid overdose were more than 6 times more likely to receive naloxone than the national average. Further studies should examine long-term outcomes, including reduction in overdose, treatment of opioid use disorder, and reduced opioid-related use of acute care.
